# Nonfatal Drug Overdoses Treated in Emergency Departments — United States, 2016–2017

**DOI:** 10.15585/mmwr.mm6913a3

**Published:** 2020-04-03

**Authors:** Alana M. Vivolo-Kantor, Brooke E. Hoots, Lawrence Scholl, Cassandra Pickens, Douglas R. Roehler, Amy Board, Desiree Mustaquim, Herschel Smith, Stephanie Snodgrass, Stephen Liu

**Affiliations:** ^1^Division of Overdose Prevention, National Center for Injury Prevention and Control, CDC; ^2^Epidemic Intelligence Service, CDC; ^3^Oak Ridge Institute for Science and Education, Oak Ridge, Tennessee.

In 2017, drug overdoses caused 70,237 deaths in the United States, a 9.6% rate increase from 2016 (*1*). Monitoring nonfatal drug overdoses treated in emergency departments (EDs) is also important to inform community prevention and response activities. Analysis of discharge data provides insights into the prevalence and trends of nonfatal drug overdoses, highlighting opportunities for public health action to prevent overdoses. Using discharge data from the Healthcare Cost and Utilization Project’s (HCUP) Nationwide Emergency Department Sample (NEDS), CDC identified nonfatal overdoses for all drugs, all opioids, nonheroin opioids, heroin, benzodiazepines, and cocaine and examined changes from 2016 to 2017, stratified by drug type and by patient, facility, and visit characteristics. In 2017, the most recent year for which population-level estimates of nonfatal overdoses can be generated, a total of 967,615 nonfatal drug overdoses were treated in EDs, an increase of 4.3% from 2016, which included 305,623 opioid-involved overdoses, a 3.1% increase from 2016. From 2016 to 2017, the nonfatal overdose rates for all drug types increased significantly except for those involving benzodiazepines. These findings highlight the importance of continued surveillance of nonfatal drug overdoses treated in EDs to inform public health actions and, working collaboratively with clinical and public safety partners, to link patients to needed recovery and treatment resources (e.g., medication-assisted treatment).

The 2017 HCUP NEDS data set is a nationally representative, stratified sample of ED visits from nonfederal, hospital-based EDs in 36 U.S. states and the District of Columbia.* Hospital discharge data represent the reference standard in nonfatal overdose surveillance and allow generation of population-level estimates to examine rate changes over time. Using 2016 and 2017 NEDS data, six drug overdose indicators were classified using *International Classification of Diseases, Tenth Revision, Clinical Modification* (ICD-10-CM) discharge diagnosis codes: 1) all-drugs, 2) all opioids, 3) nonheroin opioids, 4) heroin, 5) benzodiazepines, and 6) cocaine. All diagnosis fields were searched for initial encounter^†^ visits for intent (i.e., unintentional, intentional self-harm, assault, and undetermined).^§^ Polysubstance overdoses could be classified under multiple overdose indicators; thus, groups are not mutually exclusive.

Annual rates for drug overdose per 100,000 population were calculated by sex, age group, U.S. Census region of facility,^¶^ county urbanization level of facility,** and intent. All rates, except age group, were age-adjusted.^††^ Absolute and relative rate changes^§§^ were calculated from 2016 to 2017 by patient, facility, and visit characteristics for each overdose indicator; z-tests were used to compare changes that occurred from 2016 to 2017 and for pairwise comparisons between groups for 2017 rates, with p-values <0.05 considered statistically significant. Only selected comparisons were tested for statistical significance, and all results presented were statistically significant. Analyses were conducted using SAS (version 9.4; SAS Institute) to account for HCUP’s complex survey design and weighting.

In 2017, there were 967,615 nonfatal drug overdose ED visits (300.2 per 100,000 population) (Table 1). From 2016 to 2017, rates for nonfatal overdoses increased for those involving all drugs (4.3%), all opioids (3.1%), nonheroin opioids (3.6%), heroin (3.6%), and cocaine (32.9%), whereas the rate for overdoses involving benzodiazepines decreased 5.2% (Table 1) (Table 2) (Table 3).

**TABLE 1:** Annual number and age-adjusted rate* of emergency department visits^†^ for nonfatal overdoses involving all drugs^§^ and nonfatal overdoses involving all opioids,^¶^ by patient, facility, and visit characteristics — United States, 2016 and 2017

Characteristic	All drugs^§^						All opioids^¶^					
	2016		2017		Change from 2016 to 2017**		2016		2017		Change from 2016 to 2017**	
	No.	Rate (SE)	No.	Rate (SE)	Absolute rate change	Relative rate change	No.	Rate (SE)	No.	Rate (SE)	Absolute rate change	Relative rate change
**All**	**921,337**	**287.9 (0.304)**	**967,615**	**300.2 (0.310)**	**12.3**	**4.3^††^**	**293,900**	**90.2 (0.169)**	**305,623**	**93.0 (0.171)**	**2.8**	**3.1^††^**
**Sex**												
Male	443,132	278.5 (0.424)	469,426	292.4 (0.432)	13.9	5.0**^††^**	172,609	107.5 (0.262)	182,169	112.6 (0.268)	5.1	4.7**^††^**
Female	478,026	297.1 (0.438)	498,064	308.2 (0.445)	11.1	3.7**^††^**	121,223	72.5 (0.213)	123,428	73.1 (0.213)	0.6	0.8**^††^**
**Age group (yrs)**												
0–14	93,923	154.0 (0.503)	92,945	152.3 (0.500)	−1.7	−1.1**^††^**	3,918	6.4 (0.103)	3,721	6.1 (0.100)	−0.3	−4.7**^††^**
15–19	94,134	445.5 (1.452)	100,666	476.4 (1.501)	30.9	6.9**^††^**	8,426	39.9 (0.434)	7,541	35.7 (0.411)	−4.2	−10.5**^††^**
20–24	95,313	425.9 (1.379)	94,476	427.1 (1.390)	1.2	0.3	35,679	159.4 (0.844)	31,865	144.1 (0.807)	−15.3	−9.6**^††^**
25–34	189,474	424.1 (0.974)	202,987	447.7 (0.994)	23.6	5.6**^††^**	89,090	199.4 (0.668)	94,915	209.3 (0.679)	9.9	5.0**^††^**
35–44	130,904	323.5 (0.894)	141,605	346.4 (0.921)	22.9	7.1**^††^**	50,084	123.8 (0.553)	54,223	132.7 (0.570)	8.9	7.2**^††^**
45–54	125,147	292.5 (0.827)	127,210	300.2 (0.842)	7.7	2.6**^††^**	43,589	101.9 (0.488)	44,533	105.1 (0.498)	3.2	3.1**^††^**
55–64	99,521	240.0 (0.761)	108,543	258.5 (0.785)	18.5	7.7**^††^**	37,773	91.1 (0.469)	41,246	98.2 (0.484)	7.1	7.8**^††^**
≥65	92,921	188.7 (0.619)	99,183	195.0 (0.619)	6.3	3.3**^††^**	25,341	51.5 (0.323)	27,579	54.2 (0.327)	2.7	5.2**^††^**
**U.S. Census region^§§^**												
Northeast	162,663	293.6 (0.742)	163,785	293.6 (0.741)	0.0	0.0	66,993	120.0 (0.472)	63,742	113.0 (0.457)	−7	−5.8**^††^**
Midwest	235,882	356.7 (0.746)	250,181	378.6 (0.770)	21.9	6.1**^††^**	79,534	119.7 (0.432)	86,002	129.2 (0.449)	9.5	7.9**^††^**
South	343,134	283.0 (0.490)	358,356	292.0 (0.495)	9.0	3.2**^††^**	104,092	84.2 (0.265)	110,478	88.6 (0.271)	4.4	5.2**^††^**
West	179,658	233.5 (0.558)	195,293	252.3 (0.578)	18.8	8.1**^††^**	43,280	54.0 (0.263)	45,402	56.1 (0.267)	2.1	3.9**^††^**
**County urbanization level^¶¶^**												
Large central metro	250,565	249.5 (0.505)	284,375	278.6 (0.529)	29.1	11.7**^††^**	74,142	71.0 (0.264)	86,882	81.8 (0.282)	10.8	15.2**^††^**
Large fringe metro	202,228	257.0 (0.579)	199,486	251.8 (0.571)	−5.2	−2.0**^††^**	77,997	99.5 (0.361)	74,211	94.0 (0.350)	−5.5	−5.5**^††^**
Medium metro	214,132	323.1 (0.710)	228,701	343.2 (0.730)	20.1	6.2**^††^**	73,838	110.8 (0.416)	74,709	111.4 (0.416)	0.6	0.5
Small metro	93,891	326.6 (1.091)	92,991	322.5 (1.083)	−4.1	−1.3**^††^**	24,952	85.5 (0.556)	25,296	86.5 (0.558)	1.0	1.2
Micropolitan (nonmetro)	92,509	352.3 (1.187)	94,676	363.3 (1.210)	11.0	3.1**^††^**	25,877	97.3 (0.622)	26,256	100.4 (0.636)	3.1	3.2**^††^**
Noncore (nonmetro)	58,074	328.2 (1.409)	55,800	318.9 (1.396)	−9.3	−2.8**^††^**	12,780	69.7 (0.644)	13,414	74.5 (0.671)	4.8	6.9**^††^**
Intent***												
Unintentional	580,671	178.9 (0.238)	622,351	189.9 (0.245)	11.0	6.1**^††^**	240,919	73.8 (0.153)	258,437	78.5 (0.157)	4.7	6.4**^††^**
Intentional self-harm	283,205	91.0 (0.173)	297,540	95.4 (0.177)	4.4	4.8**^††^**	33,823	10.5 (0.058)	31,682	9.8 (0.056)	−0.7	−6.7**^††^**
Assault	2,437	0.8 (0.016)	2,072	0.7 (0.015)	−0.1	−12.5**^††^**	248	0.1 (0.005)	189	0.1 (0.004)	0.0	0.0
Undetermined	49,404	15.4 (0.070)	39,764	12.4 (0.063)	−3.0	−19.5**^††^**	17,309	5.3 (0.041)	13,533	4.1 (0.036)	−1.2	−22.6**^††^**

**Abbreviation:** SE = standard error.

* Rates are age-adjusted using the direct method and the 2000 U.S. Census standard population, except for age-specific crude rates. All rates are per 100,000 population. Statistical testing was completed using rates rounded to 1 decimal place and standard errors rounded to 3 decimal places.

^†^ Categories of nonfatal drug overdose visits are not mutually exclusive because overdose visits might involve more than one drug. Summing of categories will result in greater than the total number of visits in a year.

^§^ Nonfatal drug overdose visits are classified using the *International Classification of Diseases, Tenth Revision, Clinical Modification* (ICD–10-CM). ICD-10-CM diagnosis codes for all drugs included codes with T36-T50 with a sixth character of 1, 2, 3, or 4 (exceptions for T36.9, T37.9, T39.9, T41.4, T42.7, T43.9, T45.9, T47.9, and T49.9, which were included if the code had a fifth character of 1, 2, 3, or 4). Only codes with a seventh character of “A” (initial encounter) were included.

^¶^ ICD-10-CM diagnosis codes for all opioids included T40.0X1A–T40.0X4A, T40.1X1A–T40.1X4A, T40.2X1A–T40.2X4A, T40.3X1A–T40.3X4A, T40.4X1A–T40.4X4A, T40.601A–T40.604A, and T40.691A–T40.694A.

** Absolute rate change is the difference in rates from 2016 to 2017. Relative rate change is the absolute rate change divided by the 2016 rate, multiplied by 100. Z-tests were used to determine significance.

^††^ Statistically significant (p-value <0.05).

^§§^ Facility geographic regions were derived from U.S. Census regions: https://www.hcup-us.ahrq.gov/db/vars/hosp_region/nedsnote.jsp. *Northeast:* Connecticut, Maine, Massachusetts, New Hampshire, New Jersey, New York, Pennsylvania, Rhode Island, and Vermont. *Midwest:* Illinois, Indiana, Iowa, Kansas, Michigan, Minnesota, Missouri, Nebraska, North Dakota, Ohio, South Dakota, and Wisconsin. *South:* Alabama, Arkansas, Delaware, District of Columbia, Florida, Georgia, Kentucky, Louisiana, Maryland, Mississippi, North Carolina, Oklahoma, South Carolina, Tennessee, Texas, Virginia, and West Virginia. *West:* Alaska, Arizona, California, Colorado, Hawaii, Idaho, Montana, Nevada, New Mexico, Oregon, Utah, Washington, and Wyoming.

^¶¶^ County urbanization levels for facilities were determined using the 2013 National Center for Health Statistics Urban-Rural Classification Scheme for Counties. https://www.cdc.gov/nchs/data_access/urban_rural.htm.

*** In ICD-10-CM, the fifth or sixth character in the diagnosis code indicates intent. Possible values include accidental (unintentional), intentional self-harm, assault, undetermined intent, adverse effect, and underdosing. Adverse effect and underdosing are not applicable values for all of the different drug poisoning diagnosis codes. In this report, the intent was set to “Missing” for emergency department visits with multiple overdose intents listed.

**TABLE 2:** Annual number and age-adjusted rate* of emergency department visits^†^ for nonfatal overdoses involving nonheroin opioids^§^ and nonfatal overdoses involving heroin,^¶^ by patient, facility, and visit characteristics — United States, 2016 and 2017

Characteristic	Nonheroin opioids^§^						Heroin^¶^					
	2016		2017		Change from 2016 to 2017**		2016		2017		Change from 2016 to 2017**	
	No.	Rate (SE)	No.	Rate (SE)	Absolute rate change	Relative rate change	No.	Rate (SE)	No.	Rate (SE)	Absolute rate change	Relative rate change
**All**	**139,326**	**41.3 (0.113)**	**145,363**	**42.8 (0.115)**	**1.5**	**3.6^††^**	**147,720**	**46.9 (0.123)**	**154,626**	**48.6 (0.125)**	**1.7**	**3.6^††^**
**Sex**												
Male	68,034	41.6 (0.162)	73,113	44.5 (0.167)	2.9	7.0^††^	101,442	64.1 (0.204)	106,466	66.7 (0.207)	2.6	4.1**^††^**
Female	71,244	40.8 (0.157)	72,236	40.9 (0.156)	0.1	0.2	46,258	29.6 (0.139)	48,146	30.5 (0.141)	0.9	3.0**^††^**
**Age group (yrs)**												
0–14	3,575	5.9 (0.098)	3,480	5.7 (0.097)	−0.2	−3.4	99	0.2 (0.016)	87	0.1 (0.015)	−0.1	−50.0**^††^**
15–19	5,165	24.4 (0.340)	5,017	23.7 (0.335)	−0.7	−2.9	3,111	14.7 (0.264)	2,437	11.5 (0.234)	−3.2	−21.8**^††^**
20–24	10,350	46.2 (0.455)	10,563	47.8 (0.465)	1.6	3.5**^††^**	25,113	112.2 (0.708)	21,326	96.4 (0.660)	−15.8	−14.1**^††^**
25–34	25,869	57.9 (0.360)	28,893	63.7 (0.375)	5.8	10.0**^††^**	62,398	139.7 (0.559)	65,445	144.3 (0.564)	4.6	3.3**^††^**
35–44	20,452	50.5 (0.353)	22,342	54.7 (0.366)	4.2	8.3**^††^**	28,621	70.7 (0.418)	30,972	75.8 (0.431)	5.1	7.2**^††^**
45–54	24,631	57.6 (0.367)	23,894	56.4 (0.365)	−1.2	−2.1**^††^**	17,452	40.8 (0.309)	19,612	46.3 (0.330)	5.5	13.5**^††^**
55–64	26,607	64.2 (0.393)	27,344	65.1 (0.394)	0.9	1.4	9,367	22.6 (0.233)	12,027	28.6 (0.261)	6.0	26.5**^††^**
≥65	22,678	46.1 (0.306)	23,831	46.9 (0.304)	0.8	1.7	1,558	3.2 (0.080)	2,720	5.3 (0.103)	2.1	65.6**^††^**
**U.S. Census region^§§^**												
Northeast	23,841	41.0 (0.272)	24,048	41.1 (0.272)	0.1	0.2	42,094	77.3 (0.382)	38,797	70.5 (0.364)	−6.8	−8.8**^††^**
Midwest	32,665	47.2 (0.267)	35,244	51.2 (0.279)	4.0	8.5**^††^**	45,744	70.9 (0.336)	50,004	77.0 (0.350)	6.1	8.6**^††^**
South	55,674	43.6 (0.188)	58,171	45.1 (0.191)	1.5	3.4**^††^**	46,039	38.8 (0.183)	50,278	42.0 (0.189)	3.2	8.2**^††^**
West	27,146	33.5 (0.206)	27,899	34.0 (0.207)	0.5	1.5	13,843	17.7 (0.152)	15,547	19.7 (0.160)	2.0	11.3**^††^**
**County urbanization level^¶¶^**												
Large central metro	35,096	33.6 (0.182)	39,954	37.6 (0.191)	4.0	11.9**^††^**	36,565	35.0 (0.186)	45,025	42.5 (0.203)	7.5	21.4**^††^**
Large fringe metro	32,213	39.0 (0.221)	32,207	39.0 (0.221)	0.0	0.0	44,890	59.5 (0.283)	41,175	54.2 (0.269)	−5.3	−8.9**^††^**
Medium metro	33,229	47.8 (0.268)	36,026	51.6 (0.278)	3.8	7.9**^††^**	39,216	61.1 (0.313)	37,316	57.8 (0.304)	−3.3	−5.4**^††^**
Small metro	13,761	45.3 (0.398)	13,693	44.5 (0.392)	−0.8	−1.8	10,358	37.4 (0.375)	11,031	40.1 (0.388)	2.7	7.2**^††^**
Micropolitan (nonmetro)	14,771	52.3 (0.446)	13,435	47.9 (0.429)	−4.4	−8.4**^††^**	10,522	43.0 (0.425)	12,330	50.8 (0.463)	7.8	18.1**^††^**
Noncore (nonmetro)	8,896	45.5 (0.508)	8,588	43.8 (0.498)	−1.7	−3.7**^††^**	3,365	21.5 (0.375)	4,475	28.9 (0.437)	7.4	34.4**^††^**
Intent***												
Unintentional	103,785	30.4 (0.096)	113,392	33.1 (0.100)	2.7	8.9**^††^**	131,886	41.9 (0.117)	140,419	44.1 (0.119)	2.2	5.3**^††^**
Intentional self-harm	26,149	8.1 (0.051)	24,434	7.5 (0.049)	−0.6	−7.4**^††^**	6,700	2.1 (0.026)	6,517	2.1 (0.026)	0.0	0.0
Assault	127	0.04 (0.003)	63	0.02 (0.003)	−0.02	−50.0**^††^**	111	0.03 (0.003)	92	0.03 (0.003)	0.0	0.0
Undetermined	8,208	2.5 (0.028)	6,209	1.9 (0.024)	−0.6	−24.0**^††^**	8,447	2.7 (0.029)	6,909	2.2 (0.026)	−0.5	−18.5**^††^**

**Abbreviation:** SE = standard error.

* Rates are age-adjusted using the direct method and the 2000 U.S. Census standard population, except for age-specific crude rates. All rates are per 100,000 population. Statistical testing was completed using rates rounded to one decimal place and standard errors rounded to three decimal places.

^†^ Categories of nonfatal drug overdose visits are not mutually exclusive because overdose visits might involve more than one drug. Summing of categories will result in greater than the total number of visits in a year.

^§^ Nonfatal drug overdose visits are classified using the *International Classification of Diseases, Tenth Revision, Clinical Modification* (ICD–10-CM). ICD-10-CM diagnosis codes for nonheroin opioids included T40.0X1A–T40.0X4A, T40.2X1A–T40.2X4A, T40.3X1A–T40.3X4A, T40.4X1A–T40.4X4A, T40.601A–T40.604A, and T40.691A–T40.694A.

^¶^ ICD-10-CM diagnosis codes for heroin included T40.1X1A–T40.1X4A.

** Absolute rate change is the difference in rates from 2016 to 2017. Relative rate change is the absolute rate change divided by the 2016 rate, multiplied by 100. Z-tests were used to determine significance.

^††^ Statistically significant (p-value <0.05).

^§§^ Facility geographic regions were derived from U.S. Census regions: https://www.hcup-us.ahrq.gov/db/vars/hosp_region/nedsnote.jsp. *Northeast:* Connecticut, Maine, Massachusetts, New Hampshire, New Jersey, New York, Pennsylvania, Rhode Island, and Vermont. *Midwest:* Illinois, Indiana, Iowa, Kansas, Michigan, Minnesota, Missouri, Nebraska, North Dakota, Ohio, South Dakota, and Wisconsin. *South:* Alabama, Arkansas, Delaware, District of Columbia, Florida, Georgia, Kentucky, Louisiana, Maryland, Mississippi, North Carolina, Oklahoma, South Carolina, Tennessee, Texas, Virginia, and West Virginia. *West:* Alaska, Arizona, California, Colorado, Hawaii, Idaho, Montana, Nevada, New Mexico, Oregon, Utah, Washington, and Wyoming.

^¶¶^ County urbanization levels for facilities were determined using the 2013 National Center for Health Statistics Urban-Rural Classification Scheme for Counties. https://www.cdc.gov/nchs/data_access/urban_rural.htm.

*** In ICD-10-CM, the fifth or sixth character in the diagnosis code indicates intent. Possible values include accidental (unintentional), intentional self-harm, assault, undetermined intent, adverse effect, and underdosing. Adverse effect and underdosing are not applicable values for all of the different drug poisoning diagnosis codes. In this report, the intent was set to “Missing” for emergency department visits with multiple overdose intents listed.

**TABLE 3:** Annual number and age-adjusted rate* of emergency department visits^†^ for nonfatal overdoses involving benzodiazepines^§^ and nonfatal overdoses involving cocaine,^¶^ by patient, facility, and visit characteristics — United States, 2016 and 2017

Characteristic	Benzodiazepines^§^						Cocaine^¶^					
	2016		2017		Change from 2016 to 2017**		2016		2017		Change from 2016 to 2017**	
	No.	Rate (SE)	No.	Rate (SE)	Absolute rate change	Relative rate change	No.	Rate (SE)	No.	Rate (SE)	Absolute rate change	Relative rate change
**All**	**123,548**	**38.1 (0.110)**	**118,352**	**36.1 (0.107)**	**−2.0**	**−5.2^††^**	**27,247**	**8.5 (0.052)**	**36,919**	**11.3 (0.060)**	**2.8**	**32.9^††^**
**Sex**												
Male	50,313	31.3 (0.142)	48,218	29.7 (0.138)	−1.6	−5.1^††^	18,498	11.5 (0.086)	24,852	15.2 (0.098)	3.7	32.2**^††^**
Female	73,219	44.6 (0.168)	70,130	42.3 (0.163)	−2.3	−5.2**^††^**	8,745	5.5 (0.060)	12,052	7.5 (0.069)	2.0	36.4**^††^**
**Age group (yrs)**												
0–14	3,866	6.3 (0.102)	3,563	5.8 (0.098)	−0.5	−7.9**^††^**	129	0.2 (0.019)	160	0.3 (0.021)	0.1	50.0**^††^**
15–19	9,721	46.0 (0.467)	8,951	42.4 (0.448)	−3.6	−7.8**^††^**	689	3.3 (0.124)	876	4.1 (0.140)	0.8	24.2**^††^**
20–24	11,882	53.1 (0.487)	11,278	51.0 (0.480)	−2.1	−4.0**^††^**	2,546	11.4 (0.225)	2,857	12.9 (0.242)	1.5	13.2**^††^**
25–34	23,707	53.1 (0.345)	22,914	50.5 (0.334)	−2.6	−4.9**^††^**	6,703	15.0 (0.183)	8,903	19.6 (0.208)	4.6	30.7**^††^**
35–44	21,439	53.0 (0.362)	20,776	50.8 (0.353)	−2.2	−4.2**^††^**	5,437	13.4 (0.182)	7,132	17.4 (0.207)	4.0	29.9**^††^**
45–54	22,890	53.5 (0.354)	20,552	48.5 (0.338)	−5.0	−9.3**^††^**	6,804	15.9 (0.193)	8,687	20.5 (0.220)	4.6	28.9**^††^**
55–64	18,260	44.0 (0.326)	18,478	44.0 (0.324)	0.0	0.0	4,121	9.9 (0.155)	6,787	16.2 (0.196)	6.3	63.6**^††^**
≥65	11,783	23.9 (0.220)	11,841	23.3 (0.214)	−0.6	−2.5	816	1.7 (0.058)	1,517	3.0 (0.077)	1.3	76.5**^††^**
**U.S. Census region^§§^**												
Northeast	18,948	33.1 (0.246)	17,920	31.1 (0.238)	−2.0	−6.0**^††^**	6,892	12.3 (0.152)	8,040	14.2 (0.162)	1.9	15.4**^††^**
Midwest	29,863	45.0 (0.265)	27,706	41.4 (0.254)	−3.6	−8.0**^††^**	5,188	7.7 (0.110)	6,430	9.6 (0.123)	1.9	24.7**^††^**
South	49,807	40.6 (0.185)	48,459	39.0 (0.180)	−1.6	−3.9**^††^**	12,494	10.3 (0.094)	18,878	15.1 (0.112)	4.8	46.6**^††^**
West	24,931	32.1 (0.206)	24,267	30.9 (0.202)	−1.2	−3.7**^††^**	2,673	3.4 (0.066)	3,571	4.5 (0.076)	1.1	32.4**^††^**
**County urbanization level^¶¶^**												
Large central metro	32,154	31.6 (0.179)	34,086	33.1 (0.182)	1.5	4.7**^††^**	9,926	9.6 (0.098)	17,525	16.5 (0.127)	6.9	71.9**^††^**
Large fringe metro	27,493	34.1 (0.209)	24,013	29.5 (0.194)	−4.6	−13.5**^††^**	6,171	7.8 (0.101)	6,901	8.7 (0.107)	0.9	11.5**^††^**
Medium metro	27,875	41.6 (0.255)	29,427	43.5 (0.259)	1.9	4.6**^††^**	6,390	9.7 (0.124)	6,948	10.5 (0.129)	0.8	8.2**^††^**
Small metro	13,829	48.2 (0.421)	11,541	39.5 (0.378)	−8.7	−18.0**^††^**	1,877	6.8 (0.160)	2,051	7.4 (0.167)	0.6	8.8**^††^**
Micropolitan (nonmetro)	12,574	47.2 (0.434)	11,083	41.6 (0.408)	−5.6	−11.9**^††^**	1,418	5.6 (0.153)	1,770	7.0 (0.170)	1.4	25.0**^††^**
Noncore (nonmetro)	8,604	48.2 (0.541)	7,229	41.1 (0.503)	−7.1	−14.7**^††^**	678	4.0 (0.157)	859	5.3 (0.186)	1.3	32.5**^††^**
Intent***												
Unintentional	57,597	17.4 (0.074)	55,843	16.7 (0.072)	−0.7	−4.0**^††^**	20,758	6.4 (0.045)	30,364	9.2 (0.054)	2.8	43.8**^††^**
Intentional self-harm	57,200	17.9 (0.076)	55,583	17.3 (0.075)	−0.6	−3.4**^††^**	3,717	1.2 (0.020)	3,828	1.2 (0.020)	0.0	0.0
Assault	325	0.1 (0.006)	287	0.1 (0.006)	0.0	0.0	101	0.03 (0.003)	73	0.02 (0.003)	−0.01	−33.3**^††^**
Undetermined	7,024	2.2 (0.027)	5,286	1.6 (0.023)	−0.6	−27.3**^††^**	2,396	0.7 (0.015)	2,297	0.7 (0.015)	0.0	0.0

**Abbreviation:** SE = standard error.

* Rates are age-adjusted using the direct method and the 2000 U.S. Census standard population, except for age-specific crude rates. All rates are per 100,000 population. Statistical testing was completed using rates rounded to one decimal place and standard errors rounded to three decimal places.

^†^ Categories of nonfatal drug overdose visits are not mutually exclusive because overdose visits might involve more than one drug. Summing of categories will result in greater than the total number of visits in a year.

^§^ Nonfatal drug overdose visits are classified using the *International Classification of Diseases, Tenth Revision, Clinical Modification* (ICD–10-CM). ICD-10-CM diagnosis codes for benzodiazepines included T42.4X1A–T42.4X4A.

^¶^ ICD-10-CM diagnosis codes for cocaine included T40.5X1A–T40.5X4A.

** Absolute rate change is the difference in rates from 2016 to 2017. Relative rate change is the absolute rate change divided by the 2016 rate, multiplied by 100. Z-tests were used to determine significance.

^††^ Statistically significant (p-value <0.05).

^§§^ Facility geographic regions were derived from U.S. Census regions: https://www.hcup-us.ahrq.gov/db/vars/hosp_region/nedsnote.jsp. *Northeast:* Connecticut, Maine, Massachusetts, New Hampshire, New Jersey, New York, Pennsylvania, Rhode Island, and Vermont. *Midwest:* Illinois, Indiana, Iowa, Kansas, Michigan, Minnesota, Missouri, Nebraska, North Dakota, Ohio, South Dakota, and Wisconsin. *South:* Alabama, Arkansas, Delaware, District of Columbia, Florida, Georgia, Kentucky, Louisiana, Maryland, Mississippi, North Carolina, Oklahoma, South Carolina, Tennessee, Texas, Virginia, and West Virginia. *West:* Alaska, Arizona, California, Colorado, Hawaii, Idaho, Montana, Nevada, New Mexico, Oregon, Utah, Washington, and Wyoming.

^¶¶^ County urbanization levels for facilities were determined using the 2013 National Center for Health Statistics Urban-Rural Classification Scheme for Counties. https://www.cdc.gov/nchs/data_access/urban_rural.htm.

*** In ICD-10-CM, the fifth or sixth character in the diagnosis code indicates intent. Possible values include accidental (unintentional), intentional self-harm, assault, undetermined intent, adverse effect, and underdosing. Adverse effect and underdosing are not applicable values for all of the different drug poisoning diagnosis codes. In this report, the intent was set to “Missing” for emergency department visits with multiple overdose intents listed.

In 2017, the highest overdose rates for all drugs were among females (308.2), persons aged 15–34 years (range = 427.1–476.4), persons in the Midwest (378.6), and persons in micropolitan (nonmetro) counties (363.3) (Table 1). From 2016 to 2017, overdose rates for all drugs increased 5.0% among males and 3.7% among females. The highest overdose rates for all opioids were among males (112.6), persons aged 25–34 years (209.3), persons in the Midwest (129.2), and persons in medium metro counties (111.4). Rates for all opioid overdoses decreased 4.7% among persons aged 0–14 years, 10.5% in persons aged 15–19 years, and 9.6% among persons aged 20–24 years. In the Midwest, overdose rates for all drugs increased by 6.1% and for all opioids by 7.9%; in the South rates for all drugs and all opioids increased by 3.2% and 5.2%, respectively; and in the West by 8.1% and 3.9%, respectively. In the Northeast, the overdose rate for all drugs remained stable, and the overdose rate for all opioids decreased 5.8%.

Overdose rates for nonheroin opioids and heroin were highest among males (44.5 and 66.7, respectively), persons aged 25–34 years (63.7 and 144.3, respectively), persons in the Midwest (51.2 and 77.0, respectively), and those in medium metro counties (51.6 and 57.8, respectively) (Table 2). Increases in rates for heroin overdose were observed among males (4.1%) and females (3.0%), whereas rates for nonheroin opioid overdoses increased only among males (7.0%). Heroin overdose rates decreased 50% among persons aged 0–14 years, 21.8% among persons aged 15–19 years, and 14.1% among persons aged 20–24 years. Rates for overdoses involving nonheroin opioids and heroin increased 8.5% and 8.6% in the Midwest, respectively, and 3.4% and 8.2%, respectively, in the South. Heroin overdose rates also increased 11.3% in the West. In the Northeast, the rate for heroin-involved overdoses decreased 8.8%.

In 2017, the highest overdose rates for benzodiazepines were among females (42.3), persons aged 20–44 years (range = 50.5–51.0), persons in the Midwest (41.4), and persons in medium metro counties (43.5) (Table 3). The rates for cocaine overdoses in 2017 were highest among males (15.2), persons aged 25–34 years (19.6) and aged 45–54 years (20.5), as well as persons in the South census region (15.1) and large central metro counties (16.5). From 2016 to 2017, rates for benzodiazepine overdoses decreased 5.1% among males and 5.2% among females. Benzodiazepine overdose rates decreased among most age groups, and cocaine-involved overdoses rates increased across all age groups. All regions of the country experienced decreases in the rates of benzodiazepine overdoses and increases in the rates of cocaine overdoses.

In large central metro counties, overdose rates increased for all drugs (11.7%), all opioids (15.2%), nonheroin opioids (11.9%), heroin (21.4%), benzodiazepines (4.7%), and cocaine (71.9%) (Table 1) (Table 2) (Table 3). Most overdoses were unintentional (75% overall; range = 48% for benzodiazepines to 91% for heroin). A consistent finding across all overdose indicators, except for benzodiazepines, was that unintentional overdoses significantly increased from 2016 to 2017. Intentional self-harm overdoses increased 4.8% for all drugs but decreased 6.7% for all opioids, 7.4% for nonheroin opioids, and 3.4% for benzodiazepines.

## Discussion

In 2017, a total of 967,615 nonfatal drug overdoses were treated in U.S. EDs. From 2016 to 2017, nonfatal overdose ED visit rates increased for each drug type except benzodiazepines, for which rates decreased 5.2%. The large increase in cocaine overdose rates (32.9%) might indicate potential increase in polysubstance overdose. A previous study found that in 2016, approximately 27% of nonfatal cocaine overdoses treated in EDs also involved an opioid, and cocaine-involved overdoses with an opioid reported increased 17% from 2015 to 2016, whereas cocaine-involved overdoses without an opioid decreased 14% (*2*). Future analyses examining drug combinations could help to determine the extent to which polysubstance use affects overdose surveillance of specific drug types. In this study, rates for nonfatal unintentional overdoses were shown to increase for each drug type except benzodiazepines and for the all-drug overdose category with self-harm intent. Rates for nonfatal drug overdoses associated with intentional self-harm, assault, and undetermined intent decreased or remained stable for most overdose indicators. Results suggest a leveling of intentional drug overdoses consistent with mortality data (*3*). Continued monitoring of nonfatal drug overdoses treated in EDs is important to inform community prevention and response activities.

Changes in rates of drug overdoses varied by age group, region, and urbanization level. Decreases in rates among persons aged 15–24 years for all opioids and heroin might be due to decreases in self-reported drug use and initiation.^¶¶^Regionally, increases in overdose rates occurred for all drugs, all opioids, heroin, and cocaine in the West, Midwest, and South, which are consistent with increases in drug supply and deaths across these regions and states (*4*,*5*). For example, from 2016 to 2017, cocaine drug reports increased significantly in the South and Midwest (*4*), and cocaine-involved deaths increased in the West, Midwest, and South (*5*). The decrease in the rate for nonfatal all opioid overdoses seen in the Northeast is not consistent with drug supply reports, which increased in 2017 (*4*). However, it is possible that the lethality of opioids in the supply (e.g., illicitly manufactured fentanyl)*** might result in an increase in cases with rapid progression to death, with fewer opportunities for transport to an ED for care. Large central metro areas experienced increases in every overdose indicator; these are largely consistent with results from other data sources, including syndromic ED surveillance and mortality data from similar periods (*6*,*7*).

The findings in this report are subject to at least seven limitations. First, CDC did not assess polysubstance overdose, and it is possible that some overdoses were not classified correctly given limits of drug testing in EDs (*8*). Second, CDC could not determine whether illicit or prescribed drugs were driving some drug-specific overdose rate increases from 2016 to 2017. Third, coding practices might vary by facility and might affect the rates presented rather than actual changes in overdose prevalence. Fourth, ED visits included unique events, not unique persons, and might reflect multiple visits for one person. Fifth, these findings likely underestimated the actual prevalence of nonfatal drug overdoses because some overdoses might not be seen in EDs. Sixth, determining overdose intent in the ED setting without necessary patient context might be challenging, which might affect the accuracy of recording of intent. Finally, hospital discharge data are not as timely or localized as other data sources, including ED syndromic surveillance and emergency medical services data. Syndromic surveillance and emergency medical services data are also available at the state level and smaller geographic areas and can inform allocation of resources at a more local level. The results might not represent current trends in overdose morbidity because of the data time lag and the rapidly evolving drug market. However, they do provide more representative, comparable population estimates derived from final clinical diagnoses than do other data sources.

Overall, the increases in nonfatal overdoses suggest that enhanced surveillance, prevention, treatment, and public safety response efforts are needed to curb the increasing trends of nonfatal drug overdoses. In September 2019, CDC implemented the Overdose Data to Action (OD2A)^†††^ program, that strives to improve and expand surveillance and prevention efforts for states, territories, and localities through higher quality, more comprehensive, and more timely data on drug overdose morbidity and mortality, along with enhanced and data-driven prevention activities. With these activities, many persons who would have died from a fatal overdose are now able to receive lifesaving care, including better access to medication-assisted treatment, which might be initiated in ED settings, and subsequent linkage to care for substance use disorders and co-occurring mental disorders (*9*). In addition, implementing postoverdose protocols in EDs, including naloxone provision to patients who use opioids or other illicit drugs (*9*), checking patients’ prescription histories in prescription drug monitoring program data, and following the CDC Guideline for Prescribing Opioids for Chronic Pain when treating patients with chronic pain might prevent future overdoses (*10*).

**Summary**

**What is already known about this topic?**

In 2017, U.S. drug overdose deaths increased 9.6% from 2016. Emergency department (ED) discharge data can estimate nonfatal overdose prevalence and, because of the ability to conduct standardized analyses, track changes across time.

**What is added by this report?**

From 2016 to 2017, the nonfatal overdose ED visits rates for all drugs, all opioids, nonheroin opioids, heroin, and cocaine increased significantly, whereas those for benzodiazepines decreased significantly.

**What are the implications for public health practice?**

Using ED data to track trends in nonfatal drug overdoses is a critical strategy for expanding overdose surveillance and tailoring prevention resources to populations most affected, including initiation of medication-assisted treatment in ED settings and subsequent linkage to care for substance use disorders.

## Footnotes

* https://www.hcup-us.ahrq.gov/db/nation/neds/NEDS2017Introduction.pdf.

† https://www.cdc.gov/injury/wisqars/pdf/ICD-10-CM_External_Cause_Injury_Codes-a.pdf.

§ Intent was set to missing for ED visits with multiple overdose intents listed for any of the ICD-10-CM diagnosis codes.

¶ Facility geographic regions were derived from U.S. Census regions: https://www.hcup-us.ahrq.gov/db/vars/hosp_region/nedsnote.jsp. *Northeast:* Connecticut, Maine, Massachusetts, New Hampshire, New Jersey, New York, Pennsylvania, Rhode Island, and Vermont. *Midwest:* Illinois, Indiana, Iowa, Kansas, Michigan, Minnesota, Missouri, Nebraska, North Dakota, Ohio, South Dakota, and Wisconsin. *South:* Alabama, Arkansas, Delaware, District of Columbia, Florida, Georgia, Kentucky, Louisiana, Maryland, Mississippi, North Carolina, Oklahoma, South Carolina, Tennessee, Texas, Virginia, and West Virginia. *West:* Alaska, Arizona, California, Colorado, Hawaii, Idaho, Montana, Nevada, New Mexico, Oregon, Utah, Washington, and Wyoming.

** County urbanization levels for facilities were determined using the 2013 NCHS Urban-Rural Classification Scheme for Counties. https://www.cdc.gov/nchs/data_access/urban_rural.htm.

†† Age-adjusted rates were calculated using the 2000 U.S. Census standard population age distribution. All rates were calculated per 100,000 population. Crude rates were rounded to one decimal place before age-adjusting, and statistical testing was completed using rates rounded to one decimal place and standard errors rounded to three decimal places.

§§ Absolute rate change is the difference in rates from 2016 to 2017. Relative change is the absolute rate change divided by the 2016 rate, multiplied by 100.

¶¶ https://www.cdc.gov/drugoverdose/pdf/pubs/2019-cdc-drug-surveillance-report.pdf.

*** https://www.dea.gov/sites/default/files/2018-11/DIR-032-18%202018%20NDTA%20final%20low%20resolution.pdf.

††† https://www.cdc.gov/drugoverdose/od2a/index.html.
